# Synthesis and Comparative Study of Polyether-*b*-polybutadiene-*b*-polyether Triblock Copolymers for Use as Polyurethanes

**DOI:** 10.3390/polym15163486

**Published:** 2023-08-21

**Authors:** Pengzhi Bi, Xiuzhong Zhu, Jinbang Han, Li Tian, Wanbin Zhang

**Affiliations:** 1 State Key Laboratory of Biobased Material and Green Papermaking, Key Laboratory of Pulp and Paper Science & Technology of Ministry of Education, Faculty of Light Industry, Shandong Academy of Sciences, Qilu University of Technology, Jinan 250353, China; bipengzhi@163.com (P.B.); han1819583@163.com (J.H.); tianli20220222@163.com (L.T.); 2 Key Laboratory for Green Leather Manufacture Technology of China National Light Industry Council, Faculty of Light Industry, Shandong Academy of Sciences, Qilu University of Technology, Jinan 250353, China; 3 Key Laboratory of Auxiliary Chemistry and Technology for Chemical Industry Ministry of Education, Shaanxi Collaborative Innovation Center of Industrial Auxiliary Chemistry and Technology, Shaanxi University of Science and Technology, Xi’an 710021, China; zhangwanbin@sust.edu.cn

**Keywords:** hydroxyl-terminated polybutadiene, polyether modified, triblock copolymer, comparative study

## Abstract

In this paper, the effects of HTPBs with different main-chain microstructures on their triblock copolymers and polyurethane properties were investigated. Three polyether-modified HTPB triblock copolymers were successfully synthesized via a cationic ring-opening copolymerization reaction using three HTPBs with different microstructures prepared via three different polymerization methods as the macromolecular chain transfer agents and tetrahydrofuran (THF) and propylene oxide (PO) as the copolymerization monomers. Finally, the corresponding polyurethane elastomers were prepared using the three triblock copolymers as soft segments and toluene diisocyanate (TDI) as hard segments. The results of an analysis of the triblock copolymers showed that the triblock copolymers had lower viscosity and glass transition temperature (*T_g_*) values as the HTPB 1,2 structure content decreased, although the effect on the thermal decomposition temperature was not significant. An analysis of the polyurethane elastomers revealed that as the content of the 1,2 structure in HTPB increased, its corresponding polyurethane elastomers showed a gradual increase in breaking strength and a gradual decrease in elongation at break. In addition, PU-1 had stronger crystallization properties compared to PU-2 and PU-3. However, the differences in the microstructures of the HTPBs did not seem to have much effect on the surface properties of the polyurethane elastomers.

## 1. Introduction

Hydroxyl-terminated polybutadiene (HTPB) is a commonly used polyurethane matrix resin in defense and civil applications [1,2,3,4,5,6]. A large number of unsaturated double bonds in the backbone give the polymer excellent low-temperature resistance and flexibility [7]. However, HTPB is non-polar and, therefore, poorly compatible with polar components. As such, there have been numerous reports on modification strategies to increase the polarity of HTPB, such as main-chain epoxidation, side-group polarization, and polyester or polyether block modification [8,9,10,11,12,13,14]. Among these strategies, polyether block modification of HTPB, which refers to the preparation of triblock copolymers, is one of the more feasible routes for HTPB polarization and has been widely investigated [15,16,17]. To date, research on polyether-modified HTPB triblock copolymers has mainly focused on the effect of the type of polyether chain segment on the performance of the resulting HTPB-based copolymers; however, the effects of the HTPB microstructure, which ultimately determines the triblock copolymer performance, have not been reported systematically.

It is well known that there are three different isomeric forms of HTPB: cis-1,4-; trans-1,4-; and 1,2-polybutadiene [18]. The different isomers have significantly different properties. For example, the glass transition temperature (*T_g_*) of pure cis-1,4 polybutadiene can be as low as −106 °C, while HTPB with a high trans-1,4 content has a glass transition temperature on the order of −15 °C, good crystallinity, and properties characteristic of a plastic [19,20]. Compared with the 1,4 isomers, increasing the 1,2-polybutadiene content increases the viscosity of HTPB. It can be seen that the different microstructures of HTPB have a great influence on its own properties, and the mechanism of its influence may be explored through a reasonable molecular simulation [21]. At present, there are three main methods for the polymerization of HTPB, namely, free radical polymerization [22,23], anionic polymerization [24,25,26], and ring-opening metathesis polymerization (ROMP) [27,28]. In a previous study, our group reported in detail the differences in the structures and properties of HTPB prepared via the three polymerization methods. It was found that the contents of cis-1,4; trans-1,4; and 1,2 units within the main-chain structures of HTPB prepared via the three polymerization methods were different, which led to different glass transition temperatures and viscosities for the synthesized HTPBs [29]. As matrix resins, the differences in HTPB microstructure should directly affect the processing and application of the materials. Likewise, the different microstructures of the HTPB chain segments in the polyether blocks of the triblock copolymers will certainly affect the use of the materials, but unfortunately, there are few related studies of these effects.

**Figure 1:**
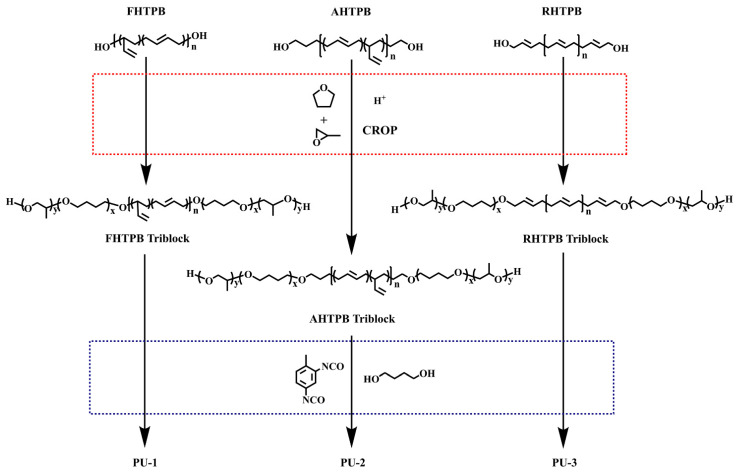
Synthesis routes of three HTPB triblock copolymers and corresponding polyurethane elastomers.

Based on the motivation discussed above, in this study, three polyether-modified HTPB triblock copolymers were designed, synthesized, and then used to prepare thermoplastic polyurethane elastomers (Figure 1). To investigate the effects of the different polybutadiene isomers on the performance of the polyether-modified HTPB triblock copolymers, three triblock copolymers with the same polyether blocks and different HTPB chain segments were prepared via free radical polymerization, anionic polymerization, and ring-opening metathesis polymerization. The HTPBs prepared via free radical polymerization, anionic polymerization, and ring-opening metathesis polymerization are referred to as FHTPB, AHTPB, and RHTPB, and the corresponding triblock copolymers are referred to as TBC_FHTPB_, TBC_AHTPB_, and TBC_RHTPB_, respectively. The molecular weights of AHTPB and RHTPB synthesized via anionic and ring-opening metathesis polymerization, respectively, were similar to those of the commercial FHTPB. Then, the three HTPBs were used as macromolecular chain transfer agents and copolymerized with THF and PO via a cationic ring-opening reaction to obtain the corresponding triblock copolymers. The molecular structures of the three triblock copolymers were characterized using FT-IR, NMR, and SEC. Subsequently, the viscosity and the thermal and processing properties of the synthesized triblock copolymers were studied using rotational viscometry, differential scanning thermal calorimetry (DSC), and thermogravimetric analysis (TGA), respectively, and compared. Finally, the TBC_FHTPB_, TBC_AHTPB_, and TBC_RHTPB_ were reacted with toluene diisocyanate (TDI) to obtain thermoplastic elastomers (named PU-1, PU-2, and PU-3, respectively), and the effects of different HTPB microstructures on the breakage strength, elongation at break, and surface properties of the corresponding elastomers were investigated via tensile testing, XRD, XPS, and contact angle measurements. We hope that the comparative study of molecular structures and properties presented here is a useful reference for the application of polyether-modified HTPB block copolymers.

## 2. Experimental Section

### 2.1. Materials

FHTPB (*M_n_* = 3400 g mol^−1^, *M_w_/M_n_* = 1.67) was kindly supplied by Qilong Chemical Corp. (Shanghai, China). 3-tert-butyldimethysilyloxy-1-propyllithium (tBDMSOPrLi) was made in the laboratory. 1,3-butadiene (15% solution in cyclohexane) was purchased from TCI (Shanghai) Development Co., Ltd., Tokyo, Japan. Cyclohexane (Cy, used immediately after drying and distilling anhydrous calcium chloride prior to use) was purchased from Sinopharm Chemical Reagent Co., Ltd., Shanghai, China. N,N-dimethylformamide (DMF) was purchased from Sinopharm Chemical Reagent Co., Ltd., Shanghai, China. Tetrabutylammonium fluoride (TBAF) was purchased from Shanghai Aladdin Biochemical Technology Co., Ltd., Shanghai, China. Methanol (MeOH) was purchased from Sinopharm Chemical Reagent Co., Ltd., Shanghai, China. Ethylene Oxide (EO) was purchased from Sinopharm Chemical Reagent Co., Ltd., Shanghai, China. Tetrahydrofuran (THF, 99.9% chromatographic purity) was purchased from Concord Technology (Tianjin) Co., Ltd., Tianjin, China. Sodium metal wire and benzophenone were added under nitrogen atmosphere before use and refluxed until the system was blue. The resulting compound was distilled, collected, and used immediately. Sodium (Na, analytical purity) was purchased from Sinopharm Chemical Reagent Co., Ltd., Shanghai, China. Cis-2-butene-1,4-diol was purchased from Shanghai Aladdin Biochemical Technology Co., Ltd., Shanghai, China. 1,5-cyclooctadiene (COD) was purchased from TCI (Shanghai) Development Co., Ltd., Tokyo, Japan. Grubbs Catalyst, 2nd Generation was purchased from Sun Chemical Technology (Shanghai) Co., Ltd., Shanghai, China. Toluene (99.5% analytical purity) was purchased from Sinopharm Chemical Reagent Co., Ltd., Shanghai, China. Sodium metal wire and benzophenone were added under nitrogen atmosphere before use and refluxed until the system was blue. The resulting compound was collected by distillation and used immediately. Propylene oxide (PO, 99.0% analytically pure, using a type 3A molecular sieve to remove water, and stored in an airtight container) was purchased from TCI (Shanghai) Development Co., Ltd., Tokyo, Japan. Boron trifluoride diethyl etherate (BF_3_·OEt_2_, 98% analytically pure) was purchased from Sun Chemical Technology (Shanghai) Co., Ltd., Shanghai, China. Dichloromethane (DCM, 99.5% analytical purity) was purchased from Tianjin Fuyu Fine Chemical Co., Ltd., Tianjin, China. Toluene diisocyanate (TDI, 98% chromatographically pure) was purchased from Shanghai Macklin Biochemical Co., Ltd., Shanghai, China. Dibutyltin dilaurate (DBTDL) was purchased from Shanghai Aladdin Biochemical Technology Co., Ltd., Shanghai, China.

### 2.2. Synthesis of HTPBs

#### 2.2.1. Synthesis of AHTPB via Living Anionic Polymerization

AHTPB was synthesized according to previous references, and the preparation process was divided into two main steps. The polymerization of 1,3-butadiene was first initiated with the monolithium initiator tBDMSOPrLi in a cyclohexane solution to obtain a mono-terminal hydroxy polybutadiene with a protecting group at the end group (tBDMSO-PB-OH). Then, the protective group was removed in a THF solution of TBAF to obtain hydroxyl-terminated polybutadiene. The preparation process was as follows: A certain amount of a 1,3-butadiene solution (15% cyclohexane solution) was added to a Schlenk tube under argon atmosphere, followed by 5 mL (2.50 mmol) of a tBDMSOPrLi solution (0.5 mol/L), and the reaction was stirred at 50 °C in an oil bath. After 5 h, 1 mL of EO was added to the reaction system as a terminator, and the reaction was continued for 2 h under argon atmosphere. Finally, 2 mL of methanol was added to the reaction system to stop the reaction, and the reaction solution was added dropwise to the methanol and left to precipitate to obtain a clear and viscous mono-terminal hydroxy polybutadiene. The reaction was stirred at room temperature for 24 h. Finally, the crude product obtained via the evaporation of THF was dissolved in DCM and extracted with water to remove impurities. Then, the final product, AHTPB, was obtained via anhydrous magnesium sulfate removal, filtration, and distillation under reduced pressure.

^1^H NMR (CDCl_3_, δ): 5.66–5.51 (-CH-C***H***=CH_2_), 5.40 (-CH_2_-C***H***=C***H***-CH_2_-), 5.09–4.86 (-CH-CH=C***H***_2_), 3.68 (-C***H***_2_-OH), 2.06 (-C***H***_2_-CH=CH-C***H***_2_-), 1.55–1.17 (-CH-C***H***_2_-).

#### 2.2.2. Synthesis of RHTPB via ROMP

RHTPB was prepared using cis-1,4-butenediol as the chain transfer agent and 1,5-cyclooctadiene as the reaction monomer. The preparation process was as follows: COD (20 g, 5.71 mmol) and 1,4-butenediol (503.10 mg, 5.71 mmol) were added to a Schlenk tube. Then, 9 mL of toluene was added as a solvent and mixed well. The reaction system was chilled for three cycles, a Grubbs II catalyst (16.6 mg dissolved in 1 mL of toluene) was added during the second cycle with nitrogen gas, and the reaction was stirred in an oil bath at 40 °C for 12 h. After the reaction, the reaction solution was added dropwise to cold methanol and placed in a refrigerator to stand, and the crude product was obtained by removing the upper layer of methanol after it was completely layered. Finally, the crude product was dissolved using DCM, and RHTPB was obtained by distillation under reduced pressure.

^1^H NMR (CDCl_3_, δ): 5.78–5.52 (-CH=C***H***-CH_2_-OH), 5.40 (-CH_2_-C***H***=C***H***-CH_2_-), 4.13 (-CH=CH-C***H***_2_-OH), 2.31–1.81 (-C***H***_2_-CH=CH-C***H***_2_-).

### 2.3. Synthesis of HTPB Triblock Copolymers

#### 2.3.1. Synthesis of FHTPB Triblock Copolymers (TBC_FHTPB_)

The polymerization reaction was carried out at a low temperature using a cationic ring-opening polymerization technique with THF and PO as the copolymerization monomers, FHTPB as the macromolecular initiator, BF_3_·OEt_2_ as the Lewis acid catalyst, and toluene as the solvent. The preparation process was as follows: First, azeotropic HTPB (30 g, 8.57 mmol) was placed in a round-bottom flask, 65 mL of toluene was added as a solvent, and the system was transferred to a low-temperature thermostatic reaction bath at 0 °C after the complete dissolution of FHTPB. THF (34.72 mL, 428.50 mmol) was added one time after 15 min using a syringe. After 15 min, the catalyst BF_3_·OEt_2_ (2.16 mL, 17.14 mmol) was added once using a syringe. After 30 min, the copolymer PO (3.00 mL, 42.85 mmol) was added drop by drop using a syringe, and the drop acceleration was controlled so that the PO was added uniformly over 40 min. After the drop addition, the reaction continued. The reaction was terminated by adding distilled water to the reaction system and left to stand. After stratification, the aqueous phase of the lower layer was aspirated out with a syringe, and the upper layer was distilled under reduced pressure to remove toluene and the small amount of remaining water. The crude product was obtained via distillation under reduced pressure until the system no longer bubbled. The crude product was dissolved with dichloromethane, poured into a beaker, washed with an equal volume of distilled water until the product was washed to neutral, and left to stand and stratify. The upper layer of water was removed, and the small amount of remaining water was removed by adding anhydrous magnesium sulfate and filtering to remove the magnesium sulfate. The filtrate was distilled under reduced pressure until no longer bubbling to obtain a colorless or light yellow product.

^1^H NMR (CDCl_3_, δ): 5.55 (-CH-C***H***=CH_2_), 5.47–5.26 (-CH_2_-C***H***=C***H***-CH_2_-), 5.02–4.87 (-CH-CH=C***H***_2_), 4.21–3.81 (-C***H***-CH=CH_2_), 3.72–3.13 (-C***H***_2_-O-), 2.04 (-C***H***_2_-CH=CH-), 1.70–1.53 (-C***H***_2_-), 1.52–1.18 (-CH-C***H***_2_-), 1.11 (-C***H***_3_).

#### 2.3.2. Synthesis of AHTPB Triblock Copolymers (TBC_AHTPB_)

TBC_AHTPB_ was prepared via the cationic ring-opening polymerization of AHTPB with THF and PO, and its synthesis and post-processing methods were similar to those of TBC_FHTPB_.

^1^H NMR (CDCl_3_, δ): 5.66–5.50 (-CH-C***H***=CH_2_), 5.38 (-CH_2_-C***H***=C***H***-CH_2_-), 5.05–4.85 (-CH-CH=C***H***_2_), 3.74–3.15 (-C***H***-CH=CH_2_, -C***H***_2_-O-), 2.05 (-C***H***_2_-CH=CH-), 1.75–1.51 (-C***H***_2_-), 1.50–1.18 (-CH-C***H***_2_-), 1.13 (-C***H***_3_).

#### 2.3.3. Synthesis of RHTPB Triblock Copolymers (TBC_RHTPB_)

TBC_RHTPB_ was prepared via cationic ring-opening polymerization between RHTPB, THF, and PO, and its synthesis and post-processing methods were similar to those of TBC_FHTPB_.

^1^H NMR (CDCl_3_, δ): 5.76–5.51 (-C***H***=C***H***-CH_2_-O-), 5.50–5.13 (-CH_2_-C***H***=C***H***-CH_2_-), 4.21–3.81 (-CH=CH-C***H***_2_-O-), 3.62–3.24 (-C***H***_2_-O-), 2.16–1.95 (-C***H***_2_-CH=CH-C***H***_2_-), 1.62 (-C***H***_2_-), 1.11 (-C***H***_3_).

### 2.4. Synthesis of Polyurethane Elastomer using HTPB Triblock Copolymers

TBC_FHTPB_, TBC_AHTPB_, and TBC_RHTPB_ were used as soft segments to synthesize PU-1, PU-2, and PU-3, respectively. All the polyurethane elastomers were prepared using a similar strategy. Take the synthesis of PU-1 as an example: TBC_FHTPB_ (2.53 g, 0.50 mmol) was placed in a round-bottom flask and 20 mL of toluene was added as a solvent. Then, TDI (0.28 g, 1.50 mmol) was added, and the reaction system was placed in an oil bath at 60 °C and stirred. After mixing well, 2 drops of a dibutyltin dilaurate catalyst were added, and the stirring of the reaction in the oil bath continued. After 6 h of stirring, BDO (90.4 mg, 1.00 mmol) was added to the reaction system, and the temperature of the oil bath was raised to 80 °C. After 12 h, the reaction was stopped, and the reaction solution was concentrated and poured into a polytetrafluoroethylene mold, which was cured to form a film to obtain the polyurethane elastomer PU-1.

### 2.5. Structural Characterization of HTPBs and HTPB Triblock Copolymers

#### 2.5.1. FT-IR Test

The FT-IR spectra of all samples were tested using a Nicolet iS10 FT-IR instrument (Nicolet Instrument Corporation, Waltham, MA, USA). The samples were prepared via the coating method, and the CH_2_Cl_2_ solution of the samples was uniformly coated on KBr, dried, and tested. The scanning area was from 4000 cm^−1^ to 500 cm^−1^.

The contents of the 1,4 unit and 1,2 unit microstructures in the HTPB backbones were calculated based on the FT-IR spectra using the following equations:

Cc%=D724cm−1KcD724cm−1Kc+D911cm−1Kv+D966cm−1Kt×100%Cv%=D724cm−1KvD724cm−1Kc+D911cm−1Kv+D966cm−1Kt×100%Ct%=D724cm−1KtD724cm−1Kc+D911cm−1Kv+D966cm−1Kt×100%

where C_c_%, C_v_%, and C_t_% are the contents of the cis-1,4 unit; 1,2 unit; and trans-1,4 unit of the microstructures in the HTPB backbone. The D values are the absorbance values of the three peaks (724 cm^−1^, 911 cm^−1^, and 967 cm^−1^) corresponding to the three different units of the HTPB backbone. The K values are the absorbance coefficients (K_c_: 31.4 mol^−1^ cm^−1^, K_v_: 151.0 mol^−1^ cm^−1^, and K_t_: 117.0 mol^−1^ cm^−1^) of the three peaks corresponding to the three different units contained in the HTPBs [24].

#### 2.5.2. NMR Test

The ^1^H NMR spectra and ^13^C NMR spectra of all samples were tested using a Bruker Avance-400 NMR instrument (Bruker Corporation, Berlin, Germany). Deuterated chloroform (CDCl_3_) was used as a solvent, and tetramethylsilane (TMS) was used as an internal reference.

#### 2.5.3. SEC-MALLS Test

The number-average molecular weight and polydispersity indexes of all samples were determined using a DAWN EOS-type gel permeation chromatography-multi-angle laser light scattering coupler (SEC-MALLS, Wyatt, Milford, MA, USA). The chromatographic column type was 5 μm 500 Å MZ-Gel SDplus 103 Å (300 × 8.0 mm). HPLC-grade THF was used as an eluent, the flow rate was 0.5 mL min-1, and the temperature was 25 °C.

### 2.6. Comparative Study of the Properties of the Triblock Copolymers and Polyurethane Elastomers

#### 2.6.1. DSC Test

The glass transition, crystalline behavior, and melting behavior of all samples were studied via DSC measurements (TA DSC250, TA Instruments, New Castle, DE, USA). The test was performed in the presence of a N_2_ atmosphere in a temperature range from −120 to 100 °C with a ramp rate of 10 °C/min. The temperature was increased to 150 °C to eliminate the thermal history of the samples before the test. Later, the temperature was reduced to −120 °C and held for 10 min.

#### 2.6.2. TGA Test

The thermal stability of all samples was studied via TGA (PerkinElmer, STA-8000, Waltham, MA, USA). The test was performed in the presence of a N_2_ atmosphere in a temperature range from 25 to 100 °C with a ramp rate of 10 °C/min.

#### 2.6.3. Viscosity Test

The viscosities of all samples were determined using a CAP2000+ Brookfield viscometer (Brookfield Corporation, New York, NY, USA). The viscometer rotated at 5.0 rpm with a temperature range of 20 to 65 °C and a temperature-controlled accuracy of ±0.2 °C.

#### 2.6.4. Tensile Test

Stress–strain curves were measured using a universal testing machine (High-speed machine VWN-20) (Kunshan Keruite Testing Instruments Corporation, Kunshan, China) at room temperature with a tensile speed of 100 mm/min.

#### 2.6.5. XRD Test

Measurements were made at 40 kV and 40 mA at room temperature with a measurement speed of 10 (°)/min and a scanning angle range of 10° to 80°.

#### 2.6.6. XPS Test

The elemental contents of the PU surfaces were measured via X-ray photoelectron spectroscopy (XPS) using an Axis Ultra Kratos instrument (Kratos Analytical Ltd., Wharfside, Manchester, UK).

#### 2.6.7. Contact Angle Test

The wetting properties of PU films were studied using water contact angle measurements.

## 3. Results and Discussion

The synthetic routes of the polyether-modified HTPB triblock copolymers is shown in Figure 1. The synthetic strategy consisted of two main steps. First, we prepared three triblock copolymers (TBC_FHTPB_, TBC_AHTPB_, and TBC_RHTPB_) via cationic ring-opening polymerization using FHTPB, AHTPB, and RHTPB as macroinitiators, respectively. Second, the corresponding polyurethane elastomers, named PU-1, PU-2, and PU-3, were prepared using the HTPB triblock copolymers as the soft segment, TDI as the hard segment, and butanediol (BDO) as the chain extender.

### 3.1. Synthesis and Characterization of TBC_FHTPB_

**Figure 2:**
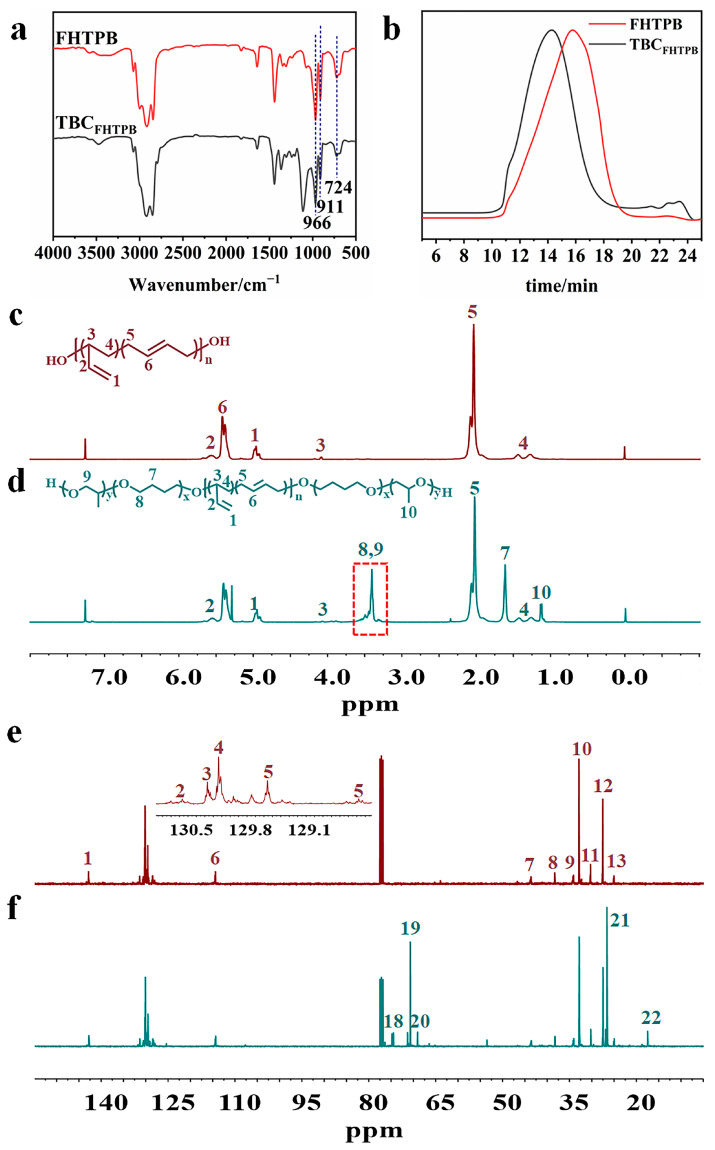
FT-IR spectra of FHTPB and TBC_FHTPB_ (**a**), SEC traces of FHTPB and TBC_FHTPB_ (**b**), ^1^H NMR of FHTPB (**c**) and TBC_FHTPB_ (**d**), and ^13^C NMR of FHTPB (**e**) and TBC_FHTPB_ (**f**).

The ^1^H NMR spectrum of TBC_FHTPB_ is shown in Figure 2d. There were peaks assigned to the methylene groups attached to the ether bond in TBC_FHTPB_ (δ = 3.72–3.13), the methylene groups in THF (δ = 1.62), and the methyl groups in PO (δ = 1.11), indicating the successful ring opening of THF with PO and confirming the successful synthesis of TBC_FHTPB_. Compared to the ^13^C NMR spectrum of pure FHTPB (Figure 2e), the spectrum for TBC_FHTPB_ had peaks assigned to the α-C and β-C in the copolyether segment at 70 ppm and 26 ppm, respectively, which further confirmed the successful synthesis of the desired triblock copolymer (as shown in Figure 2f and Table 1). The FT-IR spectrum of TBC_FHTPB_ is shown in Figure 2a, and vibrational absorption peaks from the double bonds (C=C) in the trans-1,4 isomer; cis-1,4 isomer; and 1,2 isomer were seen at 966 cm^−1^, 724 cm^−1^, and 911 cm^−1^, respectively. Compared to the FT-IR spectrum of FHTPB, the TBC_FHTPB_ spectrum also showed a clear absorption peak characteristic of the stretching vibrations of the ether bond at 1110 cm^−1^, which further confirmed the successful ring-opening polymerization reaction. In addition, the SEC trace of TBC_FHTPB_ (Figure 2b) showed a single peak that was consistent with a normal distribution, indicating that the final product contained a single homogeneous polymer. An analysis of the SEC trace gave *M_n_* = 5000 g mol^−1^ and *M_w_/M_n_* = 1.63.

### 3.2. Synthesis and Characterization of AHTPB and TBC_AHTPB_

**Figure 3:**
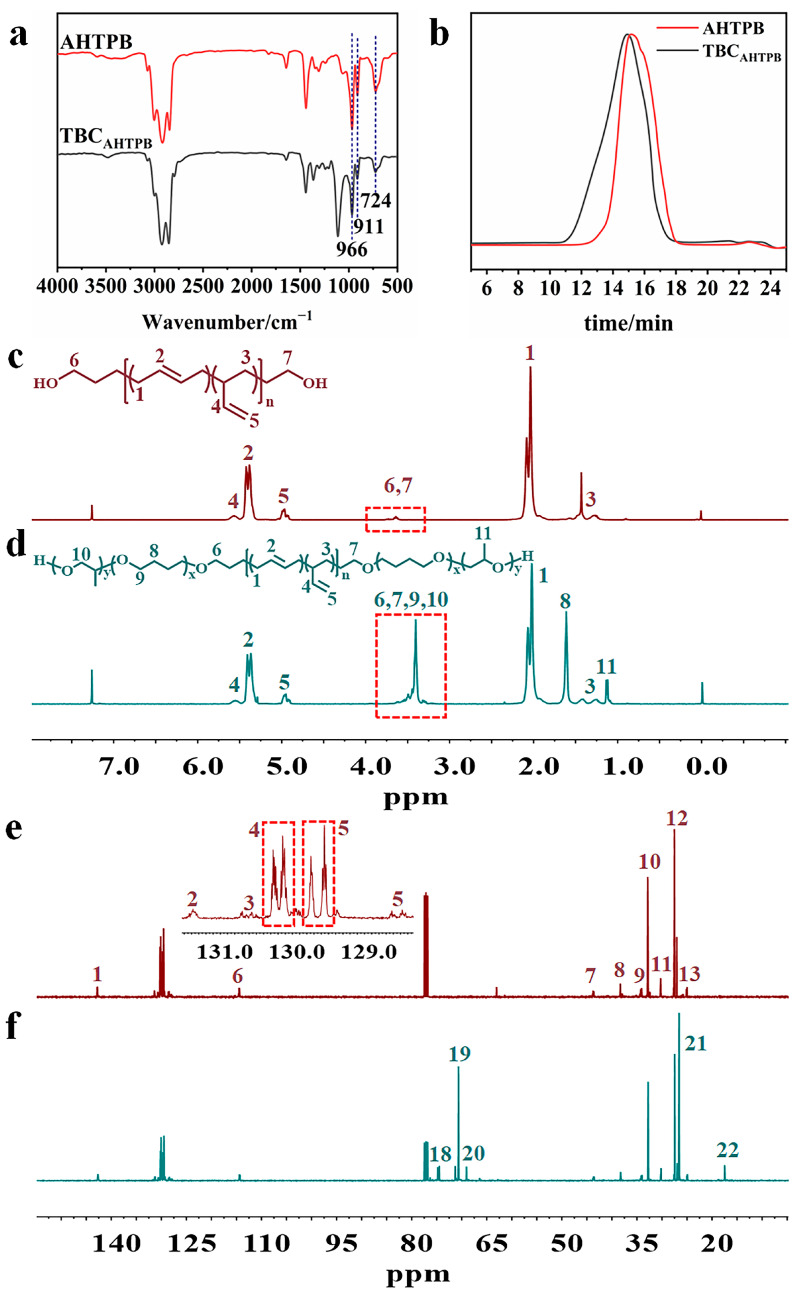
FT-IR spectra of AHTPB and TBC_AHTPB_ (**a**), SEC traces of AHTPB and TBC_AHTPB_ (**b**), ^1^H NMR of AHTPB (**c**) and TBC_AHTPB_ (**d**), and ^13^C NMR of AHTPB (**e**) and TBC_AHTPB_ (**f**).

The NMR spectrum of AHTPB is shown in Figure 3c. The peaks at δ = 5.66–5.51 were assigned to the hydrogens on the double-bonded carbon attached to the hydroxyl group of 1,2 units, the peak at δ = 5.40 was from the protons on the double-bonded carbon in the 1,4 units, the peak at δ = 5.09–4.86 was from the protons on the double-bonded terminal carbon of the 1,2 units, the peak at δ = 3.68 was from the protons on the methylene group attached to the terminal hydroxyl group, the peak at δ = 2.06 was from the protons from the methylene groups of the 1,4 units, and the peaks at δ = 1.55–1.17 were from the protons on the methylene group of the 1,2 units. The presence of these peaks in the ^1^H NMR spectrum confirmed that AHTPB was successfully synthesized. In addition, the SEC trace of AHTPB showed a single peak with *M_n_* = 3500 g mol^−1^ and *M_w_/M_n_* = 1.03 (Figure 3b). Similar to the results for TBC_FHTPB_, proton peaks from the methylene group attached to the ether bond (δ = 3.74–3.15), peaks from the protons in the methylene group in THF (δ = 1.75–1.51), and peaks from the protons in the methyl group in PO (δ = 1.13) were also present in the ^1^H NMR spectrum of TBC_AHTPB_ (Figure 3d). Peaks corresponding to the α-C and β-C in the polyether segments at 70 ppm and 26 ppm, respectively, were also observed in the ^13^C NMR spectrum of TBC_AHTPB_, confirming that TBC_AHTPB_ was successfully synthesized (Figure 3f and Table 1). Furthermore, in addition to the vibrational absorption peaks corresponding to trans-1,4 units; cis-1,4 units; and 1,2 units at 966 cm^−1^, 724 cm^−1^, and 911 cm^−1^, respectively, the FT-IR spectrum of TBC_AHTPB_ had a clear vibrational absorption peak corresponding to the ether bond stretching at 1114 cm^−1^, which further confirmed the completion of the ring-opening polymerization reaction (Figure 3a). The SEC trace of TBC_AHTPB_ showed a single sharp peak with *M_n_* = 4800 g mol^−1^ and *M_w_/M_n_* = 1.11 (Figure 3b). Notably, the molecular weight distribution of TBC_AHTPB_ was narrower than that of TBC_FHTPB_, which was attributed to the fact that the molecular weight distribution of the triblock copolymers was mainly inherited from that of HTPBs, and the molecular weight distribution of AHTPB was narrower than that of FHTPB due to the different polymerization mechanisms.

### 3.3. Synthesis and Characterization of RHTPB and TBC_RHTPB_

**Figure 4:**
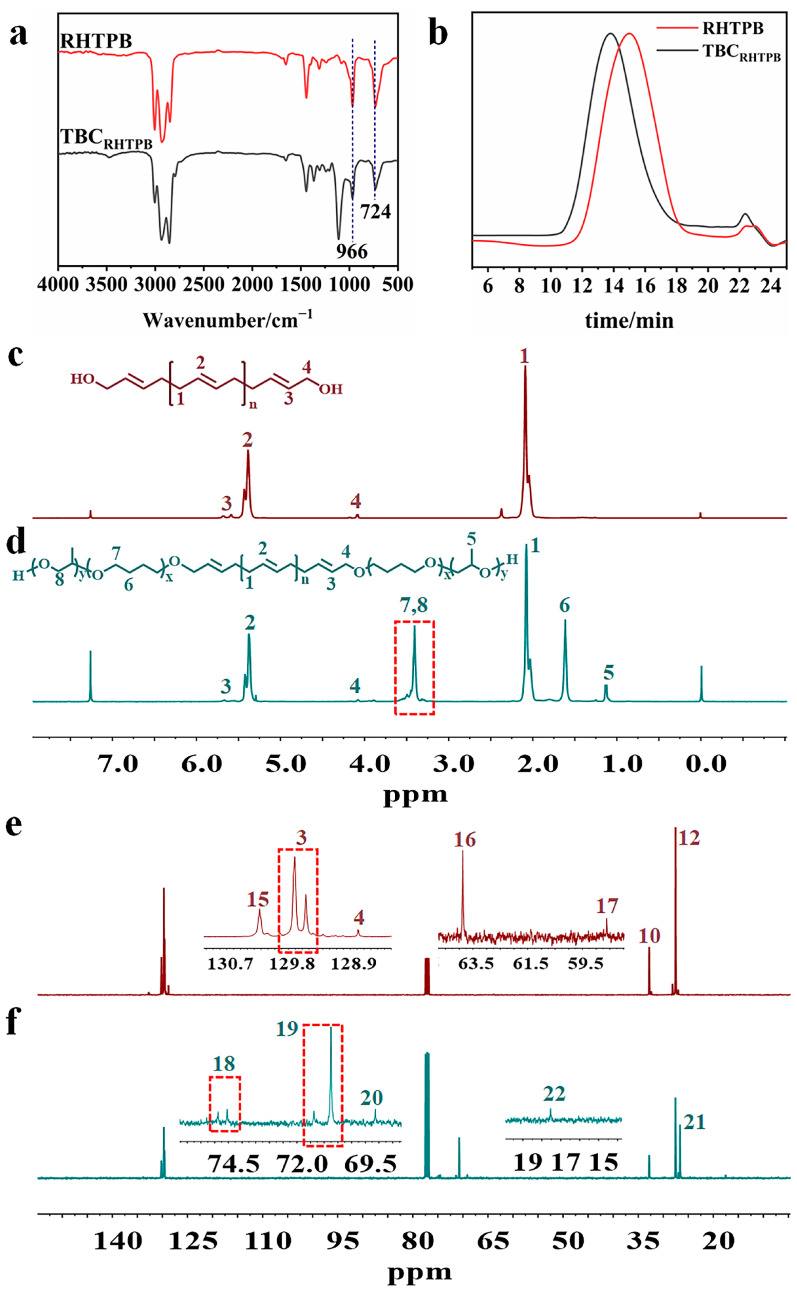
FT-IR spectra of RHTPB and TBC_RHTPB_ (**a**), SEC traces of RHTPB and TBC_RHTPB_ (**b**), ^1^H NMR of RHTPB (**c**) and TBC_RHTPB_ (**d**), and ^13^C NMR of RHTPB (**e**) and TBC_RHTPB_ (**f**).

The ^1^H NMR spectrum of RHTPB is shown in Figure 4c. The peaks at δ = 5.78–5.52 were from the protons on the double-bonded carbon attached to the hydroxymethyl groups, the peak at δ = 5.40 was the proton peak from the double bonds in the 1,4 units, the peak at δ = 4.13 was the proton peak of the methylene groups attached to the terminal hydroxyl groups, and the peaks at δ = 2.31–1.81 were the proton peaks from the methylene groups in the 1,4 units. The ^1^H NMR spectrum confirmed that RHTPB was successfully synthesized. The SEC trace of RHTPB also contained a single peak with *M_n_* = 3500 g mol^−1^ and *M_w_/M_n_* = 1.47 (Figure 4b). Similar to the two previous triblock copolymers, the peaks from the methylene groups attached to the ether bonds (δ = 3.62–3.24), the methylene groups in THF (δ = 1.62), and the methyl groups in PO (δ = 1.11) were also observed in the ^1^H NMR spectrum of TBC_RHTPB_ (Figure 4d). In addition, in the ^13^C NMR spectrum of TBC_RHTPB_, the expected peaks from the α-C and β-C in the polyether segment were also observed at 70 ppm and 26 ppm, respectively (Figure 4f and Table 1). In the FT-IR spectrum of TBC_RHTPB,_ the characteristic absorption peak of the ether bond at 1112 cm^−1^ confirmed the completion of the ring-opening polymerization reaction (Figure 4a). Significantly, the FT-IR spectrum of RHTPB only contained absorption peaks corresponding to trans-1,4 units and cis-1,4 units at 966 cm^−1^ and 724 cm^−1^, respectively, and no absorption peaks for 1,2 units at 911 cm^−1^ were observed. The difference in microstructure was attributed to the mechanism of RHTPB synthesis, resulting in the absence of 1,2 units. According to the SEC traces, TBC_RHTPB_ contained a single structure with *M_n_* = 4800 g mol^−1^ and *M_w_/M_n_* = 1.62 (Figure 4b). All the above results confirmed that TBC_RHTPB_ was successfully synthesized.

### 3.4. Viscosity of the HTPBs and Their Triblock Copolymers

**Figure 5:**
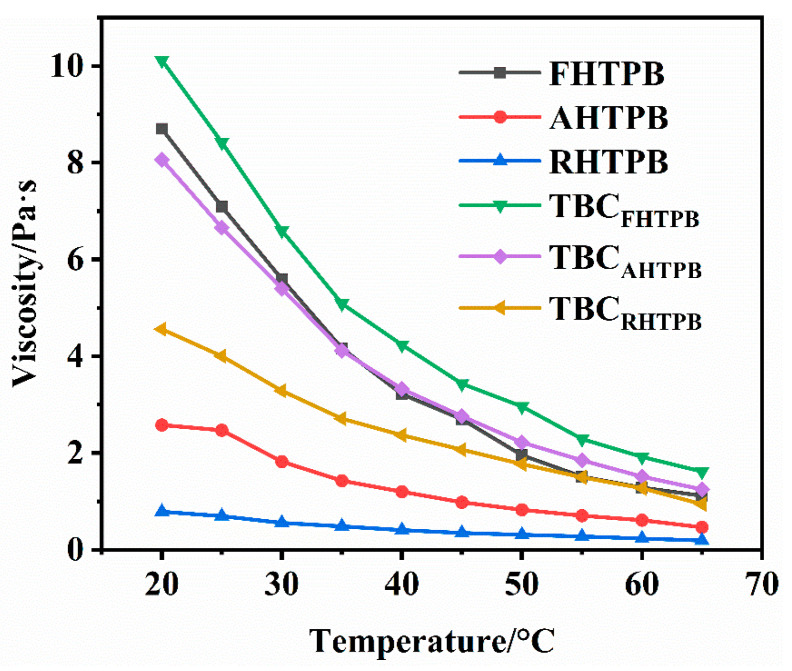
Viscosities of the FHTPB, AHTPB, RHTPB, TBC_FHTPB_, TBC_AHTPB_, and TBC_RHTPB_ as a function of temperature.

The overall content of 1,2 units in the HTPB affects the viscosity of the polymers. The viscosities of the HTPBs and the corresponding triblock copolymers at different temperatures are shown in Figure 5. The viscosities of HTPBs and their triblock copolymers decreased gradually with increasing temperature. At a given temperature, the viscosity of FHTPB was the highest, followed by AHTPB, and RHTPB had the lowest viscosity. FHTPB had the highest 1,2 isomeric content, at 30.1%, which was consistent with this polymer having the highest viscosity, while RHTPB contained no 1,2 units and therefore had the lowest viscosity (the isomeric content of each HTPB microstructure is shown in Table 2). The viscosities of the triblock copolymers showed the same trend as the corresponding HTPBs but were significantly higher than the HTPBs. The higher viscosities were attributed to the longer molecular chains and higher molecular weights of the triblock copolymers, which restricted the movements of the chain segments.

### 3.5. Thermal Properties of the HTPBs and Their Triblock Copolymers

**Figure 6:**
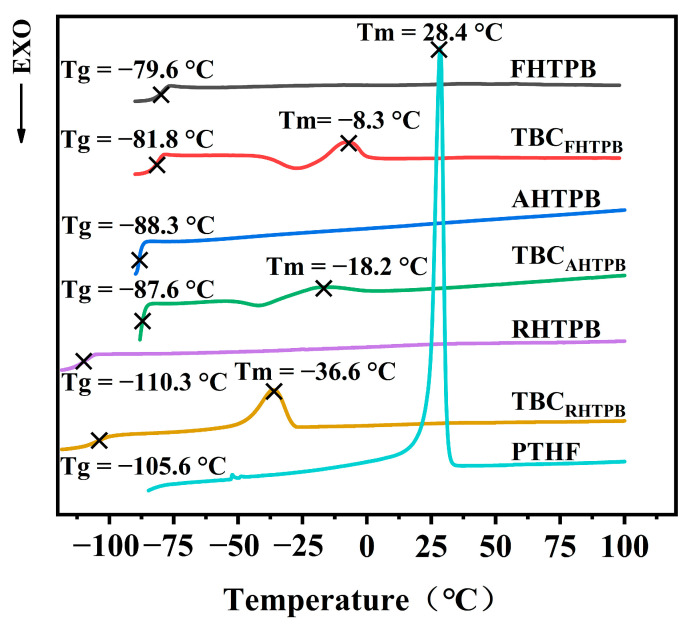
DSC heating curves of the PTHF, FHTPB, AHTPB, RHTPB, TBC_FHTPB_, TBC_AHTPB_, and TBC_RHTPB_.

The DSC heating curves of PTHF, the HTPBs, and the HTPB triblock copolymers are shown in Figure 6. FHTPB, AHTPB, and RHTPB are amorphous polymers with glass transition temperatures of −79.6 °C, −88.3 °C, and −110.3 °C, respectively. The DSC results showed that *T_g_* gradually decreased as the 1,2 content decreased. The *T_g_* values of TBC_FHTPB_, TBC_AHTPB_, and TBC_RHTPB_ were −81.8 °C, −87.6 °C, and −105.6 °C, respectively. The measured *T_g_* values of the HTPB triblock copolymers were similar to the HTPBs, indicating that the triblock copolymers inherited the *T_g_* values of the corresponding HTPBs. In addition, the DSC curves of TBC_FHTPB_, TBC_AHTPB_, and TBC_RHTPB_ showed weak melting peaks at −8.3 °C, −18.2 °C, and −36.6 °C, respectively, due to the crystallization of the polyether segments. Remarkably, the melting temperatures (*T_m_*) of the triblock copolymers were substantially lower than that of PTHF (*T_m_* = 28.4 °C). The lower *T_m_* indicated that the crystallization of the polyether segments in the triblock copolymers was constrained by the methyl side groups in the PO units, which disrupted the chain segment ordering and prevented the formation of crystal nuclei. Furthermore, the *T_g_* values of TBC_FHTPB_, TBC_AHTPB_, and TBC_RHTPB_ decreased in that order, indicating that the amorphous PB chain segments effectively inhibited the crystallization of the polyether chain segments, and the effects of the HTPB segment on the crystallization increased as the 1,2 content decreased.

**Figure 7:**
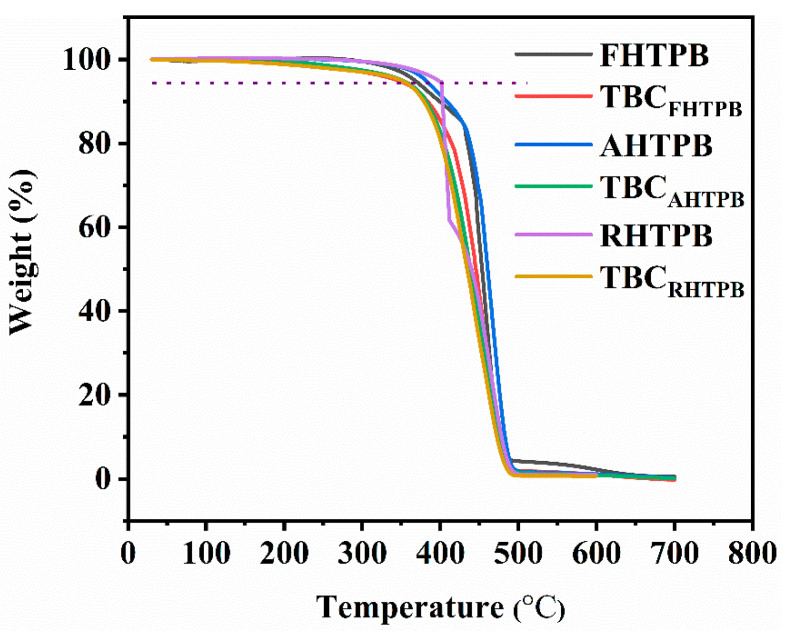
TGA curves of the FHTPB, AHTPB, RHTPB, TBC_FHTPB_, TBC_AHTPB_, and TBC_RHTPB_.

The TGA curves of the HTPBs and the triblock copolymers are shown in Figure 7. The TGA curves showed that the thermal decomposition temperatures of FHTPB, AHTPB, and RHTPB were similar and were all higher than those of the triblock copolymers. The decrease in decomposition temperature was because the polyether blocks in the triblock copolymers had a lower thermal decomposition temperature than the HTPB blocks, thus leading to a lower thermal decomposition temperature for the triblock copolymers. The thermal decomposition temperatures of TBC_FHTPB_, TBC_AHTPB_, and TBC_RHTPB_ were all approximately 357 °C, indicating that the HTPB microstructure did not affect the thermal stability of the corresponding triblock copolymers.

### 3.6. Property Comparison of Polyurethane Elastomers

#### 3.6.1. Tensile Properties

**Figure 8:**
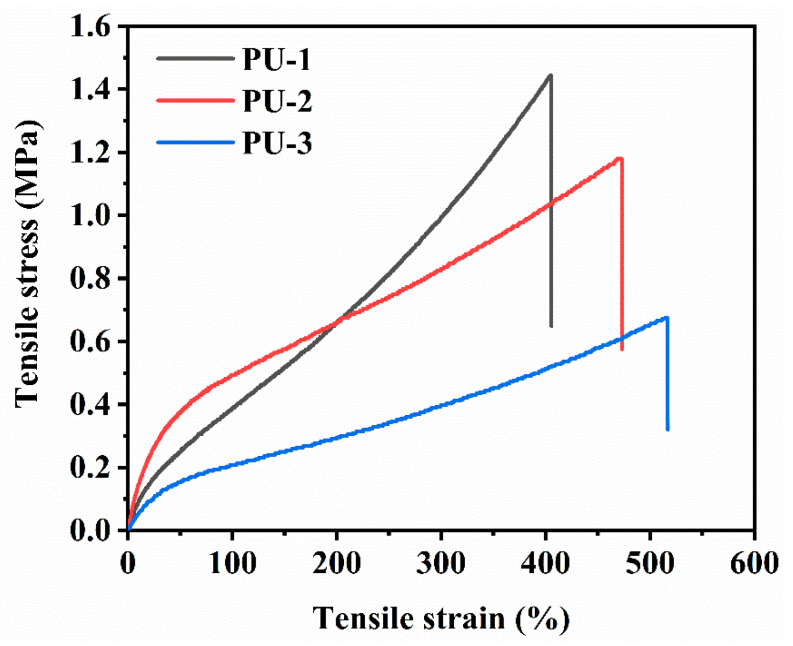
Stress–strain curves of PU-1, PU-2, and PU-3.

In order to investigate the effect of the HTPB microstructure on the performance of polyurethane elastomers prepared from the HTPB-based triblock copolymers, the breakage strength and elongation at break of the three polyurethane elastomers were tested, and the test results are shown in Figure 8. The results showed that the tensile strengths of PU-1, PU-2, and PU-3 decreased as the trans-1,4 content deceased, and their tensile strengths were 1.44 MPa, 1.18 MPa, and 0.68 MPa, respectively. Conversely, the elongations at break of PU-1, PU-2, and PU-3 increased as the cis-1,4 content increased and were 405%, 473%, and 516%, respectively. These trends showed that a high cis-1,4 content in the HTPB increased the elongation at break of the material, while a high trans-1,4 content increased the tensile strength of the material.

#### 3.6.2. XRD Analysis

**Figure 9:**
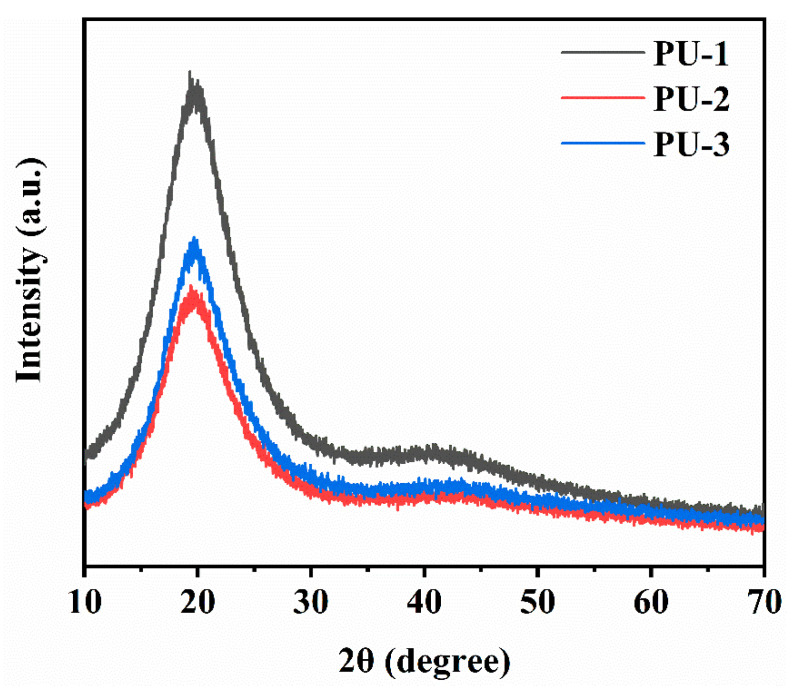
XRD curves of PU-1, PU-2, and PU-3.

The XRD curves of the polyurethane elastomers are shown in Figure 9. A broad diffraction peak was seen at 2θ = 19°~21° in the curves of PU-1, PU-2, and PU-3, which was from the hard crystalline segments in the PUs. The diffraction peaks were attributed to the structurally ordered regions of the PUs, namely, the hard segments with ordered hydrogen bonding interactions. In addition, a weaker diffraction peak at 2θ = 39°~41° was seen in the curves of PU-1, PU -2, and PU-3 and was attributed to the crystallization of the polyether block in the soft segments of the PUs. It was observed that PU-1 was more crystalline than PU-2 and PU-3, which might have been because the HTPB microstructure affected the flexibility of the chains.

#### 3.6.3. Surface Characterization of Polyurethane Elastomers

**Figure 10:**
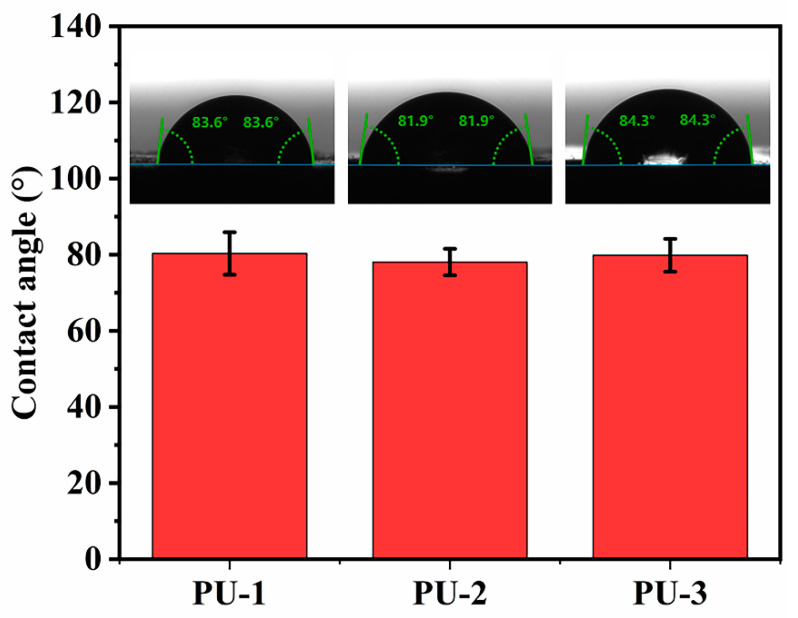
Contact angle test results of PU-1, PU-2, and PU-3.

**Figure 11:**
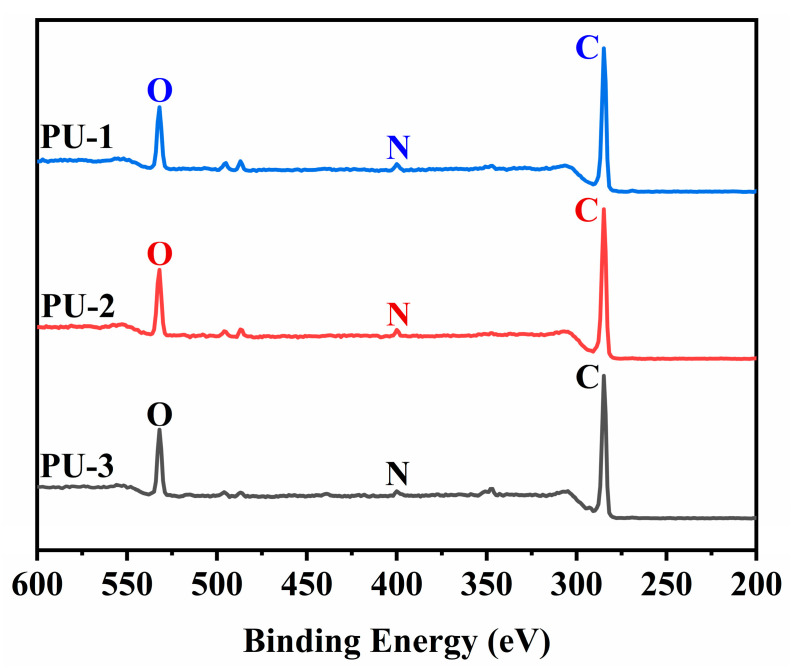
XPS test results of PU-1, PU-2, and PU-3.

The water contact angles on the PU-1, PU-2, and PU-3 surfaces are shown in Figure 10 and were found to be 83.6°, 81.9°, and 84.3°, respectively. It can be seen that the contact angles of the three PUs did not differ much, indicating that the microstructural differences of HTPB do not have much effect on the surface hydrophilicity/hydrophobicity of the PUs, and it also shows that the ratio of cis/trans isomers in HTPB does not have much effect on its own polarity. Furthermore, the XPS results clearly showed that the C, N, and O elemental contents on the surfaces of PU-1, PU-2, and PU-3 were similar, also confirming that the HTPB microstructure had little effect on the surface structure of the PUs (Figure 11).

## 4. Conclusions

We successfully synthesized AHTPB and RHTPB via reactive anion polymerization and ring-opening translocation polymerization (ROMP), respectively. To investigate the effects of different HTPB isomers on the performance of polyether-modified triblock copolymers and their elastomers, corresponding triblock copolymers, TBC_FHTPB_, TBC_AHTPB_, and TBC_RHTPB_, were synthesized via a cationic ring-opening copolymerization reaction using FHTPB, AHTPB, and RHTPB as chain transfer agents and THF and PO as copolymer monomers, respectively. The three triblock copolymers were then used to prepare three polyurethane elastomers (PU-1, PU-2, and PU-3), using the polyether-modified triblock copolymers as soft segments and TDI as hard segments. The viscosities of HTPBs and their corresponding triblock copolymers all decreased as the 1,2 unit contents decreased. In parallel, the *T_g_* values of the HTPBs gradually decreased as the contents of 1,2 units decreased and the contents of cis-1,4 units increased. The measured *T_g_* values of the corresponding triblock copolymers followed the same trend. The *T_g_* of RHTPB was −110.3 °C, and that of TBC_RHTPB_ was −105.6 °C, indicating that the introduction of polyether segments did not affect the low-temperature properties of HTPBs. In addition, TGA tests showed that the HTPB microstructure did not affect the thermal stability of the triblock copolymers, and all three triblock copolymers had a decomposition temperature of 357 °C. Studies of the polyurethane elastomer performance showed that PU-1 had the maximum tensile strength of 1.44 MPa, while PU-3 had the maximum elongation at break of 516%. The differences in the polyurethane performance were mainly attributed to the lower content of cis-1,4 units in FHTPB compared to AHTPB and RHTPB. Contact angle measurements showed that the contact angles on PU-1, PU-2, and PU-3 were similar and were around 80°. These results suggested that the microstructural content of the HTPB block did not affect the surface properties of the PUs. In conclusion, the present study clarified the effects of the different HTPB microstructures on the performances of the corresponding polyether-modified triblock copolymers and PUs through a series of comparative performance studies and provides data to support the development and application of polyether-modified HTPB block copolymers. The microstructures of polymers are key factors affecting the properties of materials. We believe the microstructure of a polymer can be a precise characterization with the development of science and technology. Meanwhile, we can obtain polymer materials with better performance by manipulating the microstructures of polymers. 

## Figures and Tables

**Table 1:** Assignment of the ^13^C NMR signals.

Peak	Chemical Shift (ppm)	Carbon
1	142.78	-$\overset{*}{C}$H=CH_2_
2	131.37	-CH_2_-CH=$\overset{*}{C}$H-CH_2_-
3, 4	130.49–130.74, 128.78–130.32	-CH_2_-$\overset{*}{C}$H=$\overset{*}{C}$H-CH_2_-
5	128.39–129.78	-CH_2_-$\overset{*}{C}$H=CH-CH_2_-
6	114.44	-CH=$\overset{*}{C}$H_2_
7	43.56	-CH_2_-$\overset{*}{C}$H-
8	38.29	-$\overset{*}{C}$H_2_-CH=CH-CH_2_-
9	34.15	-$\overset{*}{C}$H_2_-CH(C_2_H_4_)-$\overset{*}{C}$H_2_-CH=
10, 12	32.71, 27.44	-$\overset{*}{C}$H_2_-CH=CH-$\overset{*}{C}$H_2_-
11, 13	30.15, 24.95	-CH_2_-CH=CH-$\overset{*}{C}$H_2_-
14, 15	132.59, 130.22	-CH_2_-CH=$\overset{*}{C}$H-CH_2_-OH
16, 17	63.87, 58.61	-CH_2_-CH=CH-$\overset{*}{C}$H_2_-OH
18	74.39–74.85	-CH_2_-O-$\overset{*}{C}$H-CH_3_
19	70.71–71.28	-O-$\overset{*}{C}$H_2_-CH_2_-CH_2_-$\overset{*}{C}$H_2_-O-
20	69.07	-$\overset{*}{C}$H_2_-O-CH-CH_3_
21	26.52–26.61	-O-CH_2_-$\overset{*}{C}$H_2_-$\overset{*}{C}$H_2_-CH_2_-O-
22	17.48	-CH_2_-O-CH-$\overset{*}{C}$H_3_

**Table 2:** Microstructures of the FHTPB, AHTPB, and RHTPB.

Polymer	*M_n_*/g mol^−1^	*M_w_/M_n_*	% of Microstructure Determined via FT-IR		
			Cis-1,4	1,2	Trans-1,4
FHTPB	3400	1.67	26.2	30.1	43.7
AHTPB	3500	1.03	58.9	15.7	25.4
RHTPB	3500	1.47	78.0	0	22.0

## Data Availability

Not applicable.

## References

[1] Selvakumar S., Rao G.S., Reddy K.A. (2021). Diffusion of Labile Chemical Species in HTPB and HTPB-XT Solid Propellants and Its Effect over Solid Rocket Motor Properties on Aging—A Study. Propell. Explos. Pyrot..

[2] Chaturvedi S., Dave P.N. (2019). Solid propellants: AP/HTPB composite propellants. Arab. J. Chem..

[3] Li J., Ning Z., Yang W., Yang B., Zeng Y. (2022). Hydroxyl-Terminated Polybutadiene-Based Polyurethane with Self-Healing and Reprocessing Capabilities. ACS Omega.

[4] Ma L., Zhu X., Zhang W., Zhang H., Wang J., Qu J. (2021). Study on the preparation and performance comparison of side-chain hydroxyl-terminated polybutadiene derivatives with narrowly molecular weight distribution used for polyurethane. Polym. Test..

[5] Sikder B.K., Jana T. (2018). Effect of Solvent and Functionality on the Physical Properties of Hydroxyl-Terminated Polybutadiene (HTPB)-Based Polyurethane. ACS Omega.

[6] Hosseini S.R., Nikje M.M.A. (2023). Synthesis and characterization of novel epoxy-urethane coating and its graphene nanocomposites. Polym. Compos..

[7] Zhang W., Zhang T., Liu H., Zheng Y., Zhong Y., Wang G., Zhu Q., Liu X., Zhang L., Li H. (2022). Synthesis and characterization of a novel hydroxy telechelic polyfluoroether to enhance the properties of HTPB solid propellant binders. Colloids Surf. A.

[8] Zhang P., Tan W., Zhang X., Chen J., Yuan J., Deng J. (2021). Chemical Modification of Hydroxyl-Terminated Polybutadiene and Its Application in Composite Propellants. Ind. Eng. Chem. Res..

[9] Yuan B., Wang G., Tian W., Zhou L., Li C. (2023). Fabrication of Hydroxy-Terminated Polybutadiene with Piezoelectric Property by Functionalized Branch Chain Modification. Molecules.

[10] Zhang Y., Zheng J., Zhang X., Du Y., Li K., Yu G., Jia Y., Liu Y. (2021). Effect of chemical copolymerization and mixed chain extenders on mechanical properties of HTPB polyurethane. IOP Conf. Ser. Mater. Sci. Eng..

[11] Liang J., Nie J., Zhang H., Guo X., Yan S., Han M. (2023). Interaction Mechanism of Composite Propellant Components under Heating Conditions. Polymers.

[12] Liu J., Yang D., Li S., Bai C., Tu C., Zhu F., Xin W., Li G., Luo Y. (2023). Synthesis and characterization of hydroxyl-terminated polybutadiene modified low temperature adaptive self-matting waterborne polyurethane. RSC Adv..

[13] Lemos M.F., Mendes L.C., Bohn M.A. (2021). On the functionalization and characterization of hydroxyl-terminated polybutadiene with octyl-1-azide and the evaluation of polyurethane elastomers based on such modified HTPB. J. Appl. Polym. Sci..

[14] Kumar D., Mohammad S.A., Kumar A., Mane S.R., Banerjee S. (2022). An amino acid-derived ABCBA-type antifouling biohybrid with multi-stimuli responsivity and contaminant removal capability. Polym. Chem..

[15] Li L., Peng W., Liu L., Zheng S. (2022). Toughening of epoxy by nanostructures with ABA triblock copolymers: An influence of organosilicon modification of block copolymer. Polym. Eng. Sci..

[16] Wang Z., Zhao Y., Wei Y. (2022). Syntheses and properties of tri- and multi-block copolymers consisting of polybutadiene and polylactide segments. RSC Adv..

[17] Min X., Fan X. (2019). A New Strategy for the Synthesis of Hydroxyl Terminated Polystyrene-b-Polybutadiene-b-Polystyrene Triblock Copolymer with High Cis-1, 4 Content. Polymers.

[18] Dossi E., Earnshaw J., Ellison L., Santos G.R.D., Cavaye H., Cleaver D.G. (2021). Understanding and controlling the glass transition of HTPB oligomers. Polym. Chem..

[19] Garraza A.L.R., Mansilla M.A., Depaoli E.L., Macchi C., Cerveny S., Marzocca A.J., Somoza A. (2016). Comparative study of thermal, mechanical and structural properties of polybutadiene rubber isomers vulcanized using peroxide. Polym. Test..

[20] Wrana C., Schawe J.E.K. (2020). Isothermal crystallization of cis-1.4-polybutadiene at low temperatures. Thermochim. Acta.

[21] Eslami H., Gharibi A., Plathe F.M. (2021). Mechanisms of Nucleation and Solid−Solid-Phase Transitions in Triblock Janus Assemblies. J. Chem. Theory Comput..

[22] Sadeghi G.M.M., Morshedian J., Barikani M. (2006). The effect of solvent on the microstructure, nature of hydroxyl end groups and kinetics of polymerization reaction in synthesize of hydroxyl terminated polybutadiene. React. Funct. Polym..

[23] Grishchenko V.K., Boiko V.P., Svistova E.I., Yatsimirskaya T.S., Valuev V.I., Dmitrieva T.S. (1992). Hydrogen-Peroxide-Initiated Polymerization of Isoprene in Alcohol Solutions. J. Appl. Polym. Sci..

[24] Chen J., Lu Z., Pan G., Qi Y., Yi J., Bai H. (2010). Synthesis of hydroxyl-terminated polybutadiene possessing high content of 1,4-units via anionic polymerization. Chin. J. Polym. Sci..

[25] Min X., Fan X., Liu J. (2018). Utilization of steric hindrance of alkyl lithium-based initiator to synthesize high 1,4 unit- containing hydroxyl- terminated polybutadiene. R. Soc. Open Sci..

[26] Zhu X., Fan X., Zhao N., Min X., Liu J., Wang Z. (2017). Influence of mono-lithium based initiators with different steric volumes on 1,4 unit content of hydroxyl terminated polybutadiene using anionic polymerization. RSC Adv..

[27] Li L., Zhang C., Zheng S. (2017). Synthesis of POSS-terminated polycyclooctadiene telechelics via ring-opening metathesis polymerization. J. Polym. Sci. Part A Polym. Chem..

[28] Torres-Rocha O.L., Wu X., Zhu C., Crudden C.M., Cunningham M.F. (2019). Polymerization-Induced Self-Assembly (PISA) of 1,5-Cyclooctadiene Using Ring Opening Metathesis Polymerization. Macromol. Rapid Commun..

[29] Zhu X., Fan X., Zhao N., Liu J., Min X., Wang Z. (2018). Comparative study of structures and properties of HTPBs synthesized via three different polymerization methods. Polym. Test..

